# Interrupted E2F1-*miR-34c*-SCF negative feedback loop by hyper-methylation promotes colorectal cancer cell proliferation

**DOI:** 10.1042/BSR20150290

**Published:** 2016-02-03

**Authors:** Shu Yang, Bo Wu, Haimei Sun, Fengqing Ji, Tingyi Sun, Yan Zhao, Deshan Zhou

**Affiliations:** * Department of Histology and Embryology, School of Basic Medical Sciences, Capital Medical University, Beijing 100069, P.R. China; † Beijing Key Laboratory of Cancer Invasion and Metastasis Research, Beijing 100069, P.R. China; ‡ Cancer Institute of Capital Medical University, Beijing 100069, P.R. China; § Clinical Research Centre for Autoimmune Liver Disease, Beijing YouAn Hospital, Capital Medical University, Beijing 100069, China

**Keywords:** colorectal cancer, E2F1, hyper-methylation, *miR-34c*, p53, stem cell factor (SCF)

## Abstract

Tumour suppressor *miR-34c* deficiency resulted from hyper-methylation in its promoter is believed to be one of the main causes of colorectal cancer (CRC). Till date, *miR-34c* has been validated as a direct target of p53; but previous evidence suggested other transcription factor(s) must be involved in *miR-34c* transcription. In the present study, we in the first place identified a core promoter region (−1118 to −883 bp) of *pre-miR-34c* which was embedded within a hyper-methylated CpG island. Secondly, E2F1 promoted *miR-34c* transcription by physical interaction with the *miR-34c* promoter at site −897 to −889 bp. The transcriptional activating effect of E2F1 on *miR-34c* was in a p53 independent manner but profoundly promoted in the presence of p53 with exposure to 5-aza-2′-deoxycytidine (DAC). Thirdly, stem cell factor (SCF), a *miR-34c* target, was specifically reduced upon an introduction of E2F1 which lead to suppression of CRC cell proliferation. The E2F1-suppressed cell proliferation was partially abrogated by additional *miR-34c* inhibitor, indicating that the anti-proliferation effect of E2F1 was probably through activating *miR-34c*-SCF axis. Finally, SCF/KIT signalling increased E2F1 production by reducing its proteosomal degradation dependent on PI3K/Akt-GSK3β pathway. In conclusion, our results suggested the existence of E2F1-*miR-34c*-SCF negative feedback loop which was interrupted by the hyper-methylation of *miR-34c* promoter in CRC cells and increased cell proliferation.

## Abstract (precis)

E2F1 promoted miR-34c transcription which reduced its target stem cell factor (SCF) and inhibited colorectal cancer (CRC) cell proliferation. While, SCF increased E2F1 production, suggesting an existence of E2F1-miR-34c-SCF negative feedback loop, which was interrupted by hyper-methylation of miR-34c promoter in CRC cells.

## INTRODUCTION

Colorectal cancer (CRC) ranks the top five most malignant neoplasms both in China and western countries. The tumorigenesis and development of CRC are resulted from a network of multiple aberrant molecules including miRNAs. miRNAs post-transcriptionally regulate their target genes via either translational repression or mRNA degradation. Depending on their targets, miRNAs could serve as either oncogenes or tumour suppressing genes [1,2]. *miR-34* family including *miR-34a*, *miR-34b* and *miR-34c* have been suggested to be candidates of tumour suppressing genes that could induce apoptosis and cell-cycle arrest and inhibit proliferation and colony formation in soft agar [3]; whereas in CRC tissues *miR-34s* were silenced [4–6]. The anti-CRC role of *miR-34s* was probably due to down-regulating their targets, such as oncogenes *Snail1*, *Bcl2*, *E2F3*, *Sirt1* and *KIT* [7–10]. *miR-34s* are encoded by two different genes: *miR-34a* is encoded by its own transcript on chromosome 1, whereas *miR-34b* and *miR-34c* share a common primary transcript on chromosome 11 [3]. Among the three members, *miR-34a* has been studied most since the expression of *miR-34a* is at least 100-fold higher than that of *miR-34b* and *miR-34c* in CRC cells [3,10,11]. While, on the flip side, it might hint that *miR-34b/c* are more deeply associated with CRC because their expressions are pretty much inhibited than *miR-34a* in CRC. However, very few publications were focused on *miR-34b/c* in CRC. Recently, we have demonstrated that over-expression of *miR-34c* induced apoptosis and inhibited proliferation in CRC cells by silencing its target, stem cell factor (SCF, also known as KITLG) [12], suggesting *miR-34c* as a prominent target for the treatment of CRC patients.

Interests in how come *miR-34c* is down-regulated in CRC have been highlighted recent years. Till date, *miR-34c* has been verified as a direct p53 target [13,14]. Two p53-responsive elements, p53RE1 and p53RE2, are identified approximately 3 kb upstream of *miR-34c* coding sequence [13]. Activation of p53 by adriamycin (ADR) induced *miR-34c* expression in wild-type HCT-116 cells to some degree; but no such increase was detected in *p53^−/−^* HCT-116 cells [14]. Notably, Toyota et al. [14] observed a remarkable up-regulation of *miR-34c* by 5-aza-2′-deoxycytidine (DAC) treatment and a synergistic effect upon p53 activation following DAC, elucidating the fact that aberrant DNA methylation represses p53-facilitated *miR-34c* transcription. However, as indicated by Toyota et al. [14], the increase in *miR-34c* by ADR was much weaker than that by DAC; and the locus of p53-responsive elements were far from the hyper-methylated CpG island located within the *miR-34c* promoter. Thus, it inspired us to find out if there is other transcription factor(s) that could bind to the *cis*-element(s) just within the CpG island of *miR-34c* promoter and could be more involved in *miR-34c* transcription in CRC cells.

## MATERIALS AND METHODS

### Cell culture and treatment

Human CRC cell lines HT-29 (*p53^+^*) and HCT-116 (*p53^−^*) as well as 293T cell line were purchased from the Cell Bank of Chinese Academy of Sciences. All cell lines were cultured in Dulbecco's modified Eagle's medium (DMEM) medium (Life Technologies, USA) supplemented with 10% FBS (Life Technologies) and 1% penicillin/streptomycin (Life Technologies). Cells were grown at 37°C in the presence of 5% CO_2_ and treated with 5 or 10 μM DAC (Sigma) for 6 days. The medium and DAC were replaced every 24 h. DMSO (Sigma) was used as control.

### Bioinformatics

The transcriptional start site (TSS) of *miR-34c* was predicted using miRStart (http://mirstart.mbc.nctu.edu.tw/). The putative promoter sequence of *miR-34c* was retrieved from UCSC Genome Brower (http://www.genome.ucsc.edu/). Sequence conservation was analysed with Evolutionary Conserved Region Brower (http://ecrbrowser.dcode.org/). Prediction of transcription factors for *miR-34c* was conducted using TFSearch (http://www.cbrc.jp/research/db/TFSEARCH.html), NSite (http://linux1.softberry.com/) and Alggen (http://alggen.lsi.upc.edu/). CpG island was predicted by EMBOSS Cpgplot (http://www.ebi.ac.uk/Tools/seqstats/emboss_cpgplot/).

### Construction of plasmids

To construct the *miR-34c* promoter-luciferase reporter plasmids pGL3-883, pGL3-1118 and pGL3-1381, different lengths of *miR-34c* promoter were amplified from human genomic DNA by PCR and subsequently cloned into BglII and HindIII sites of pGL3-basic plasmid (Promega). *miR-34c* promoter-luciferase reporter plasmids containing site-specific mutagenesis for E2F1- or Sp1-binding site were generated using QuikChange Lightning Site-Directed Mutagenesis Kit (Agilent Technologies). To construct the E2F1 over-expression plasmid pE2F1, the open reading frame (ORF) of *E2F1* (NM_005225.2) was amplified from human genomic DNA by PCR and subsequently cloned into XhoI site of pENTER plasmid (Vigene). The primers used in aforementioned construction or mutation are listed in Supplementary Table S1.

### Plasmid transfection and dual-luciferase reporter assay

293T cells were seeded on 96-well plates. *miR-34c* promoter-luciferase reporter plasmid was co-transfected with Renilla luciferase expression vector, pRL-TK (Promega), using Lipofectamine™ 2000 (Life Technologies), according to the manufacturers’ instruction. For co-transfection, pE2F1 was co-transfected with *miR-34c* promoter-luciferase reporter plasmid and pRL-TK. Twenty-four hours after transfection, the Firefly and Renilla luciferase activities were measured using Dual Luciferase Assay System (Promega) in Multiskan FC (Thermo Scientific), according to the manufacturers’ protocols. The ratio of Firefly/Renilla activity was calculated. All luciferase assays were performed in six times.

### Methylation-specific PCR

The genomic DNA of CRC cells was extracted using QIAamp DNA Mini Kit (Qiagen). Two hundred to five hundred nanograms DNA was subject to bisulfite conversion using EZ DNA Methylation-Gold™ Kit (Zymo Research). The methylation-sensitive PCR was performed using Platinum Taq DNA Polymerase (Life Technologies). The PCR reaction consisted of an initial incubation at 94°C for 2 min, followed by 35 cycles of 94°C for 30 s, 55°C for 30 s and 68°C for 1 min using verity 96-well thermo cycler (Applied Biosystems). The primers for methylation-specific PCR (MSP) detection were designed by on-line MethPrimer tool (http://www.urogene.org/cgi-bin/methprimer/ethprimer.cgi) and are listed in Supplementary Table S1. The PCR products were electrophoresed in 0.75% agarose gel and visualized by uitraviolet illumination.

### ChIP

ChIP was performed according to the instructions of EZ-Magna ChIP™ A Kit (Merk-Millipore). Chromatin was immunoprecipitated for 24 h at 4°C using anti-E2F1 or anti-Sp1 monoclonal antibody (Merk-Millipore). 1/100 of the solution collected before adding the antibody was used as an internal control for the amount of input DNA. As a negative control, the antibody or DNA was omitted or replaced with normal rabbit IgG. Real-time PCR was carried out using SYBR Green PCR Master Mix (Life Technologies), consisting of 10 min at 94°C and 50 cycles of 20 s at 94°C and 1 min at 60°C. Fluorescent signals were detected using ABI 7500 real-time PCR system (Applied Biosystems). Primers are shown in Supplementary Table S1. Results were normalized to input expression. All PCR reactions were performed in triplicates.

### EMSA and gel shift assays

Nuclear protein extracts from CRC cells were prepared with nuclear and cytoplasmic extraction reagents (Applygen). EMSA was performed using LightShift Chemiluminescent EMSA Kit (Thermo Scientific) according to the manufacturer's protocol. The 5′-biotin-labelled probe, unlabelled probe and unlabelled mutant probe (Supplementary Table S1) were synthesized in Shanghai Sangon Biological Engineering Technology and Services Company (Shanghai). In supershift assay, anti-E2F1 monoclonal antibody (Merk-Millipore) was pre-incubated with nuclear protein extracts for 5 min at 25°C. The biotin-labelled DNA–protein complex was detected using streptavidin–horseradish peroxidase (HRP) conjugate and LightShift chemiluminescent substrate, viewed in Fusion FX Vilber Lourmat.

### Up- and down-regulation of E2F1

To up-regulate E2F1, pE2F1 was transfected into CRC cells, using Lipofectamine™ 2000. pENTER vector was used as control. To down-regulate E2F1, siRNA, siE2F1 consisting of 21 bp of oligonucleotides specifically targeting *E2F1*, and its negative control, siE2F1-NC, were purchased from Shanghai Sangon Biological Engineering Technology and Services Company (Shanghai). Transfection of 100 nM siRNA was carried out using Lipofectamine RNAiMAX Reagent (Life Technologies) according to the manufacturers’ instruction.

### RNA extraction and real-time PCR

Total miRNA was extracted using miRNApure Mini Kit (CWBiotech), according to the manufacturer's instruction. Reverse transcription was performed using Taqman microRNA RT Kit (Life Technologies) and Taqman microRNA Assay with specific stem-loop primers (Life Technologies). Real-time PCR was performed using Taqman Universal Master Mix II (Life Technologies). The reactions were incubated at 95°C for 10 min, followed by 40 cycles of 95°C for 15 s and 60°C for 1 min using ABI 7500 real-time PCR system. Results were normalized to the internal control, *RNU6B*.

Total RNA was extracted using TRIzol reagent (Life Technologies) according to the manufacturer's instruction. Reverse transcription reactions were performed using High Capacity RNA-to-cDNA Kit (Life Technologies). Real-time PCR was performed in ABI 7500 real-time PCR system using SYBR Green PCR Master Mix (Life Technologies). The primers used are shown in Supplementary Table S1. The reactions were incubated at 95°C for 10 min, followed by 40 cycles of 95°C for 15 s and 60°C for 1 min. *GAPDH* was used as internal control.

All reverse transcription reactions included no-template controls, and all PCR reactions were run in triplicates. Relative miRNA or mRNA expression was determined using the comparative C_T_ (2^−ΔΔCt^) method.

### Western blot

The harvested cells were suspended in RIPA lysis buffer (Applygen). After 10% SDS/PAGE, the proteins were transferred on to PVDF membrane (Merk-Millipore) and blocked with 5% non-fat dry milk or 5% BSA (Sigma) for 1 h. The membrane was incubated with mouse anti-SCF (1:400, Abcam), mouse anti-E2F1 (1:1000, Merk-Millipore), rabbit anti-Akt (1:1000, Cell Signaling Technology), rabbit anti-p-Akt (1:2000, Cell Signaling Technology), rabbit anti-GSK3β (1:1000, Cell Signaling Technology), rabbit anti-p-GSK3β (1:1000, Cell Signaling Technology), mouse anti-β-actin (1:4000, Santa Cruz) or rabbit anti-α-tubulin (1:1000, Santa Cruz) primary antibody at 4°C overnight. Then, the membrane was incubated with HRP-conjugated secondary goat anti-mouse IgG (1:4000, Santa Cruz) or goat anti-rabbit IgG (1:2000, Abcam) for 1 h. The proteins were detected using ECL chemiluminescence (Thermo Scientific) and viewed in Fusion FX Vilber Lourmat.

### Real-time monitoring of cellular proliferation

We employed the ‘xCELLigence’ system (ACEA Biosciences) to monitor CRC cell proliferation as previously described [12]. Briefly, cells were seeded in E-plates at a density of 8000 cells/well and transfected with plasmid and/or siRNA at standard incubator condition. Cells were resumed monitoring till cell growth hit a plateau.

### *miR-34c* loss-of-function experiment

For specific gene knockdown on *miR-34c*, *miR-34c* inhibitor and its negative control *miR-34c* inhibitor NC were purchased from Ribobio CO., LTD. *miR-34c* inhibitor or inhibitor NC was transfected into HT-29 cells using riboFECT™ CP Transfection Kit (Ribobio) according to the manufacturer's instructions.

### Activation and inactivation of SCF/KIT signalling

To activate the SCF/KIT signalling, we constructed lentivirus vector containing CDS of *KIT* gene by the technical support from GeneChem (Shanghai, China) and transfected it into HT-29 cells. After stably over-expressing KIT, exogenous rhSCF (50 ng/ml, R&D) was added into medium for 36 h. To inactivate the SCF/KIT signalling, 2 μM Imatinib (BioVision) was used for 36 h.

### Statistics

The results were presented as the mean ± S.E.M. and analysed using a student's *t* test or one-way ANOVA with the SPSS 13.0 software. A *P*-value of 0.05 or less was considered statistically significant.

## RESULTS

### Identification of *miR-34c* promoter

**Figure 1:**
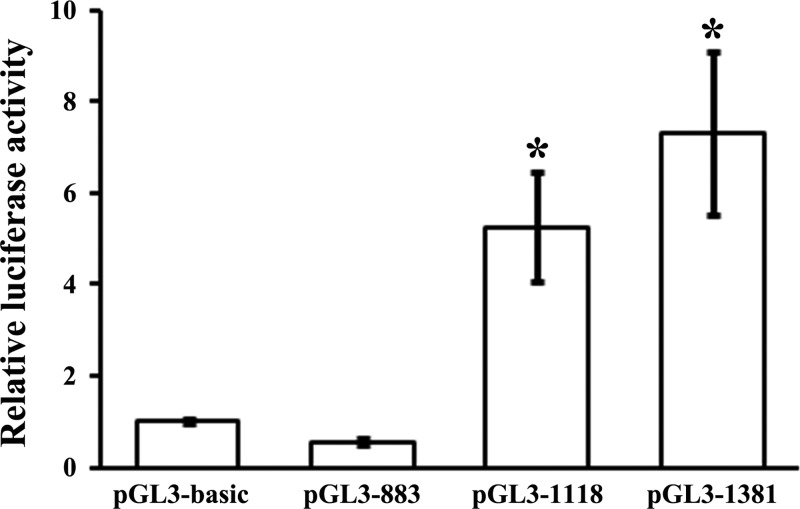
Identification of *miR-34c* promoter by luciferase assays The luciferase reporter constructs termed as pGL3-883, pGL3-1118 and pGL3-1381 as well as the negative control, pGL3-basic plasmid was each transfected into 293T cells. The dual luciferase assays were performed 24 h after transfection. The relative luciferase activity of each construct was evaluated as relative to the activity of pGL3-basic plasmid, which was arbitrarily set to 1. Transfection of pGL3-1118 and pGL3-1381 produced significantly higher luciferase activities. All luciferase assays were performed in six times (*P<0.05 compared with either pGL3-basic and pGL3-883).

*miR-34c* is an intergenetic miRNA. Unlike intragenetic miRNAs that share common TSS with their host genes and whose TSSs can be easily identified, the TSS of *pri-miR-34c* has not been identified yet. Here, using the publicly accessible algorithm MiRStart, we predicted the site at −960 bp upstream of *pre-miR-34c* as the putative TSS. A 3 kb region upstream of *pre-miR-34c* from UCSC Genome Brower was retrieved as the putative promoter. We further predicted a typical CpG island at −1076 to −245 bp upstream of *pre-miR-34c*, which embraced the putative TSS. Combined with the sequence conservation data, three luciferase reporter constructs containing decreasing lengths (883, 1118 and 1381 bp) of the putative promoter region were generated in order to experimentally verify this putative promoter region (Supplementary Figure S1). The three luciferase reporter constructs and pGL3-basic plasmid were each transfected into 293T cells to evaluate the basal promoter activity. pGL3-1118 and pGL3-1381 exhibited significantly higher luciferase activities than pGL3-883 did (*P*<0.05), bolstering that the core promoter of *pri-miR-34c* was located within −1118 to −883-bp region (236 bp) containing the TSS at −960 bp upstream of the *pre-miR-34c* (Figure 1). The experimental data matched our bioinformatics prediction and indicated that the 1118 bp region upstream of the *pre-miR-34c* had the ability to trigger *miR-34c* transcription.

### Role of CpG island methylation in *miR-34c* expression

**Figure 2:**
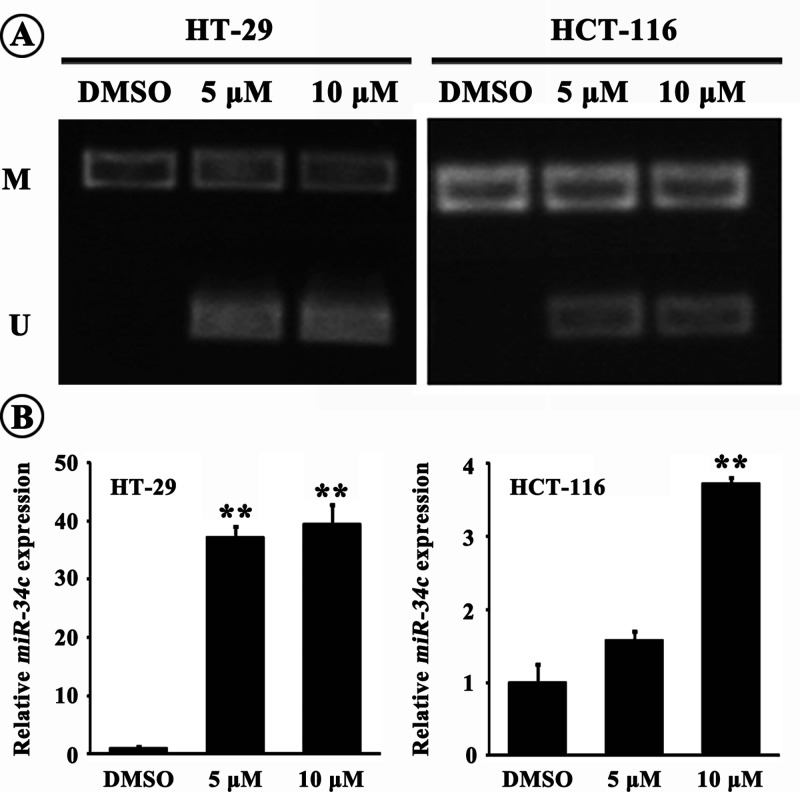
Role of promoter methylation in *miR-34c* expression (**A**) The CpG island within the *miR-34c* promoter region was hyper-methylated in CRC cells. DAC treatment induced partial demethylation indicated by the appearance of unmethylation bands (M: methylation, U: unmethylation). (**B**) DAC treatment elicited remarkable increase in *miR-34c* expression, which was much more tremendous in HT-29 cells (**P<0.01 compared with control).

Since the CpG island is within the *miR-34c* promoter, we evaluated the role of methylation state of CpG island in *miR-34c* expression using methylation and unmethylation primers specifically to the CpG island, following 6 day-DAC exposure. MSP results showed that the CpG island was hyper-methylated in CRC cells; and the 5 or 10 μM-DAC treatment induced partial demyethylation which was more apparent in *p53^+^* HT-29 cells indicated by brighter PCR bands compared with *p53^−^* HCT-116 cells (Figure 2A). Concomitantly, *miR-34c* level in CRC cells was increased after exposure to 5 or 10 μM-DAC, with higher rise in HT-29 cells (37.6-folds by 5 μM DAC or 39.3-folds by 10 μM DAC relative to control; *P*<0.01) than that in HCT-116 cells (3.7-folds by 10 μM DAC relative to control; *P*<0.01) (Figure 2B). In the following experiments, we used 10 μM DAC to treat CRC cells. The results suggested that the hyper-methylation of CpG island within the *miR-34c* promoter did repress *miR-34c* transcription probably via blocking transcription factor(s)’ binding to the *cis*-element in the CpG island. Moreover, It was consolidated that p53 did contribute to *miR-34c* transcription and probably exerted a synergic effect with other transcription factor(s).

### Identification of transcription factor binding sites in *miR-34c* promoter

**Figure 3:**
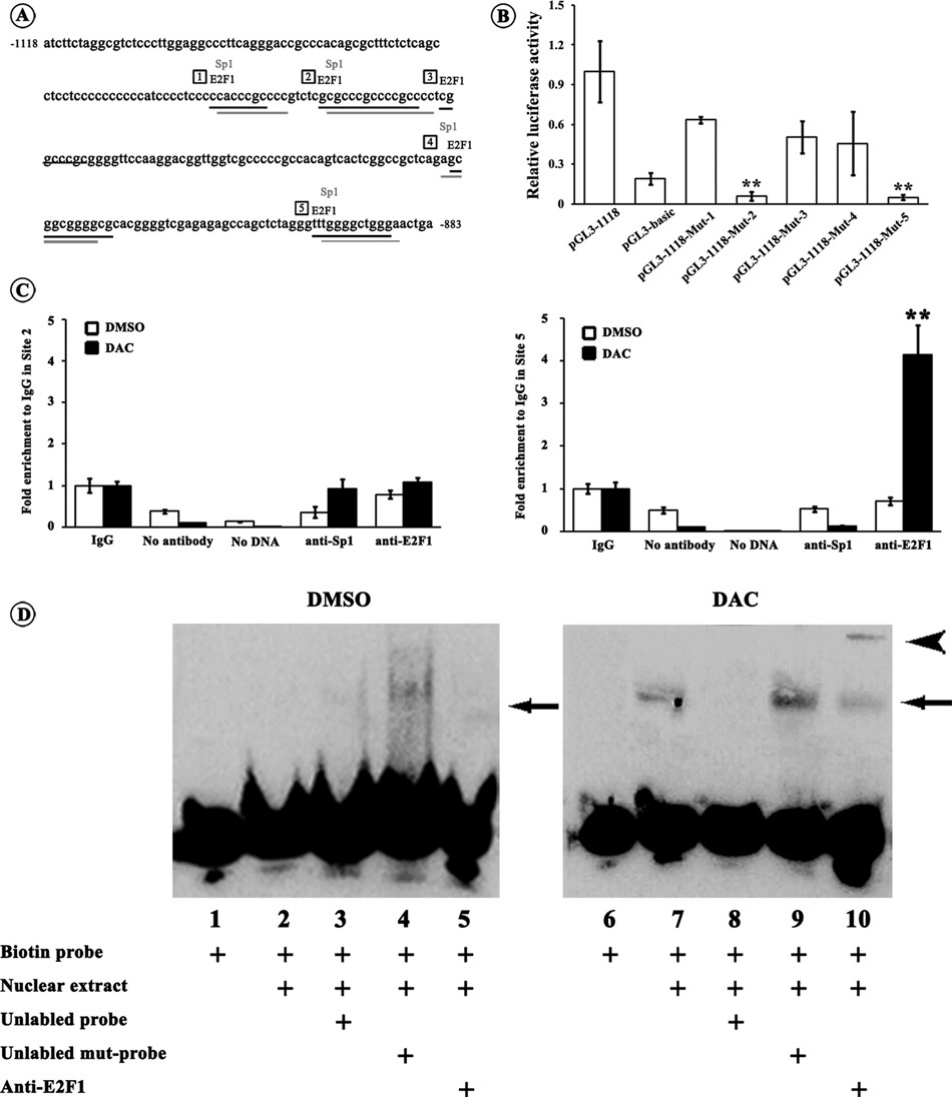
Identification of transcription factor binding sites in *miR-34c* promoter (**A**) Sequence of the *miR-34c* promoter flanking from −1118 to −1381 bp and potential E2F1- and Sp1-binding sites within it. E2F1 and Sp1 shares common binding sites in the *miR-34c* promoter; and we set Site 1–Site 5 for site-directed mutagenesis assays. Numbers in frames indicates the five binding sites. (**B**) Each pGL3-1118 mutant (Mut-1–Mut-5) luciferase reporter construct or pGL3-1118 construct was transfected into 293T cells. Twenty-four hours after the transfection, the dual luciferase assays were performed. When compared with pGL3-1118 construct, the relative luciferase activities of pGL3-1118-Mut-2 and pGL3-1118-Mut-5 constructs were significantly lowered to a level of pGL3-basic plasmid. All luciferase assays were performed in six times (**P<0.01). (**C**) HT-29 cells were treated with DAC or DMSO followed by ChIP assays. Chromatin was immunoprecipitated with the anti-E2F1 or anti-Sp1 antibody. The real-time PCR specific to Site 2 and Site 5 were performed. For the control, no antibody or no DNA was used. The values were shown relative to IgG group which was set to 1. Only the binding activity of E2F1 to Site 5 was evidently enhanced upon the DAC treatment. All PCR reactions were performed in triplicates (**P<0.01). (**D**) Nuclear extract from HT-29 cells treated with DAC or DMSO were incubated with biotin-labelled probe corresponding to putative E2F1 binding Site 5 in the *miR-34c* promoter (lanes 2–5 and 7–10). For competition system, 200-fold excess of unlabelled probe or unlabelled mutational probe was additionally added (lanes 3, 4, 8 and 9). For supershift assay, anti-E2F1 antibody was included (lanes 5 and 10). The negative control was used in the absence of nuclear extracts (lanes 1 and 6). It clearly showed the specific binding of E2F1 to the Site 5 (−897 to −889 bp) in the *miR-34c* promoter after DAC induced demethylation. The arrow indicates the specific DNA–protein complex and the arrowhead indicates the supershift.

Next, we looked forward to identifying the key transcriptional factor(s) that could regulate *miR-34c* transcription in CRC cells. Using the on-line transcription factor prediction programmes, we found that the CpG island in the −1118 to −883 bp region of the *miR-34c* promoter harboured crucial transcriptional regulatory elements, including potential E2F1- and Sp1-binding sites (Figure 3A). We used site-directed mutagenesis to abolish each binding site and performed luciferase assays. Compared with pGL3-1181, the luciferase activities of pGL3-1118-Mut-2 and pGL3-1118-Mut-5 constructs were strikingly decreased by 93.8% (*P*<0.01) and 94.8% (*P*<0.01) respectively; whereas the luciferase activities of pGL3-1118-Mut-1, pGL3-1118-Mut-3 and pGL3-1118-Mut-4 were unvarying (Figure 3B). It was suggested that the potential E2F1-binding sites at Site 2 (−1016 to −1003 bp) and Site 5 (−897 to −889 bp) and the Sp1-binding sites at Site 2 (−1015 to −1001 bp) and Site 5 (−895 to −888 bp) were probably responsible for enhancing *miR-34c* transcription. The binding properties of E2F1 and Sp1 at Site 2 and Site 5 were further validated by ChIP assays with or without DAC treatment. Immunoprecipitation of the DAC treated CRC cells with anti-E2F1 or anti-Sp1 antibody was detected by real-time PCR. Four-fold enrichment in Site 5 with anti-E2F1 antibody was observed in HT-29 cells upon the DAC treatment when compared with control (*P*<0.01; Figure 3C). But neither significant increase in Site 5 with anti-Sp1 antibody nor in Site 2 with anti-E2F1 or anti-Sp1 antibody was detected (Figure 3C). The ChIP results were further confirmed by EMSA and supershift assays. DNA probes corresponding to E2F1 binding Site 5 containing the putative E2F1 binding sequence and its 5′ and 3′ flanking regions were used. After incubation with nuclear extracts from HT-29 cells that were not treated with DAC, no obvious retarded bands were observed (Figure 3D, lanes 1–5). However, Incubation of biotin-labelled probe with nuclear extracts from DAC treated HT-29 cells resulted in a clear retarded band (Figure 3D, lane 7). A 200-fold excess of unlabelled probe prevented labelled probe from bind to the nuclear extracts (Figure 3D, lane 8), which was abrogated by unlabelled mutational probe (Figure 3D, lane 9). Furthermore, incubation of anti-E2F1 antibody caused a supershift band (Figure 3D, lane 10), indicating the presence of E2F1 in the DNA–protein-binding complex. The same results of ChIP and EMSA were obtained from HCT-116 cells (results not shown). These results provided evidences that the transcription factor E2F1 could recruit to the −897 to −889 bp region of the *miR-34c* promoter in CRC cells.

### E2F1 drives *miR-34c* transcription

**Figure 4:**
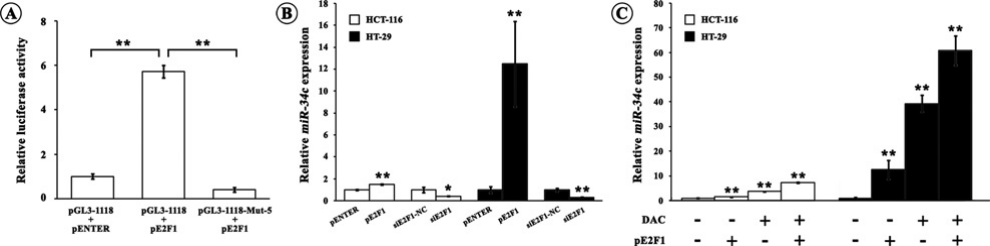
Transcriptional regulation of E2F1 on *miR-34c* (**A**) E2F1-mediated transcriptional regulation of *miR-34c* via binding to the site −897 to −889 bp. 293T cells were transfected with pGL3-1118 or pGL3-1118-Mut-5 constructs whose binding site for E2F1 at −897 to −889 bp was mutated, in the presence of pE2F1 or its control pENTER. The relative luciferase activity of pGL3-1118 construct was remarkably increased after co-transfection with pE2F1. Whereas the luciferase activity of pGL3-1118-Mut-5 construct was not varied even co-transfected with pE2F1. All luciferase assays were performed in six times (**P<0.01). (**B**) The endogenous *miR-34c* levels in CRC cells were determined after transfection of either pE2F1 or siE2F1. Over-expression of E2F1 promoted *miR-34c* expression whereas down-regulation of E2F1 decreased *miR-34c* expression. All PCR reactions were performed in triplicates (*P<0.05, **P<0.01). (**C**) DAC treatment augmented the effect of E2F1 on increasing *miR-34c* expression. All PCR reactions were performed in triplicates (**P<0.01).

To clarify the role of E2F1 in *miR-34c* transcription, we co-transfected the pGL3-1118 construct with either the E2F1 over-expression plasmid pE2F1 or the control plasmid pENTER into 293T cells. As shown in Figure 4A, the co-transfection of pGL3-1118 and pE2F1 overtly raised the luciferase activity by 5.7-folds compared with the co-transfection of pGL3-1118 with pENTER (*P*<0.01). The stimulating effect of pE2F1 on the *miR-34c* transcription was abrogated when pE2F1 was co-transfected with pGL3-1118-Mut-5 which was devoid of E2F1-binding site (*P*<0.01). The endogenous *miR-34c* was significantly increased by E2F1 over-expression in HCT-116 (1.5-folds, *P*<0.01) and HT-29 cells (12.5-folds, *P*<0.01) (Figure 4B). In contrast, E2F1 knockdown by its specific siRNA significantly reduced the endogenous *miR-34c* expression in HCT-116 (59.0%, *P*<0.05) and HT-29 cells (67.9%, *P*<0.01) (Figure 4B). Additionally, DAC treatment amplified the E2F1-induced *miR-34c* expression in HCT-116 (7.33-folds, *P*<0.01) and HT-29 cells (60.8-folds, *P*<0.01) (Figure 4C). Again, E2F1 over-expression in the presence or in the absence of DAC drove more increase in *miR-34c* in *p53^+^* HT-29 cells than that in *p53^−^* HCT-116 cells (Figures 4B and 4C).

### E2F1 decreases SCF and cell proliferation partially through increasing *miR-34c*

**Figure 5:**
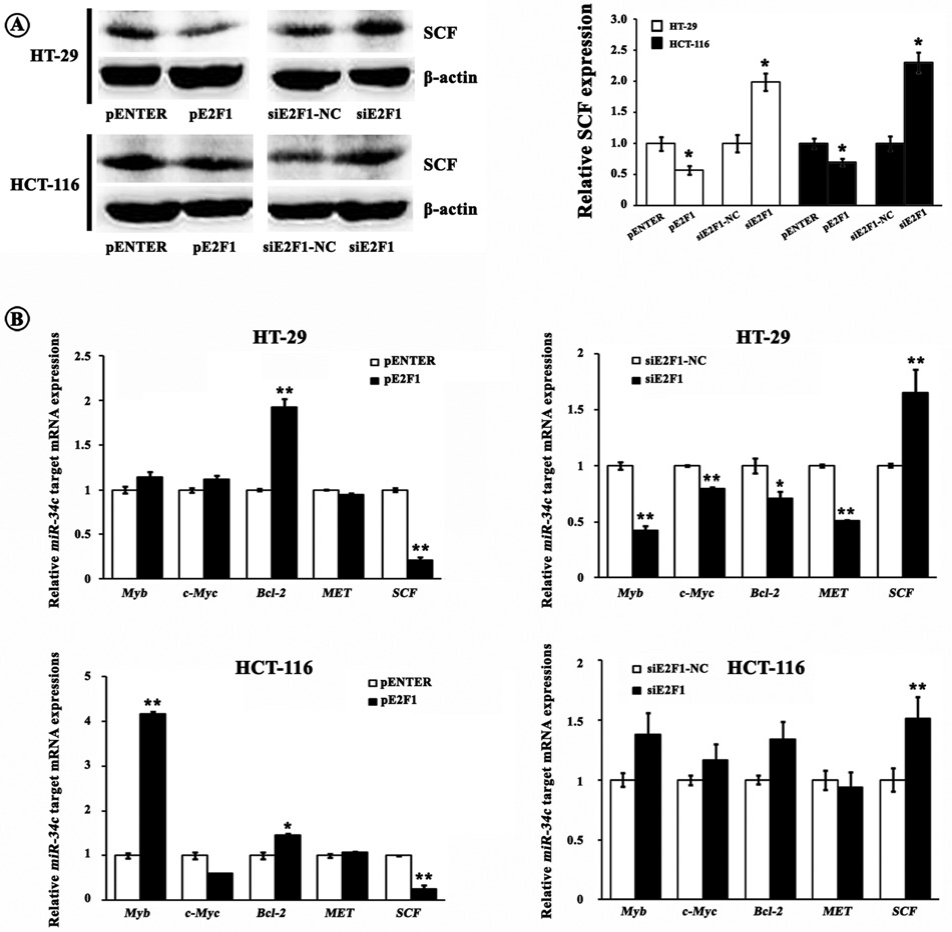
Contribution of E2F1 to expressions of *miR-34c* targets (**A**) E2F1 down-regulated the SCF protein expression. Left, western blotting bands; right, grey value analysis (*P<0.05). (**B**) E2F1 suppressed the SCF mRNA expression. However, none of the previously identified *miR-34c* targets including *Myb*, *c-Myc*, *Bcl2*, *MET* and *E2F3* were negatively regulated by E2F1 (*P<0.05, **P<0.01).

We have elucidated that *miR-34c* inhibited CRC cell proliferation via silencing its target SCF which could trigger cell proliferation by activating KIT [12]. Therefore, we asked whether E2F1 could affect SCF expression and cell proliferation since E2F1 was crucial for *miR-34c* transcription as we demonstrated above. As shown in Figures 5A and 5B, SCF mRNA and protein levels were decreased after up-regulating E2F1; whereas increased after down-regulating E2F1. Moreover, we detected some other previously identified *miR-34c* targets including *Myb*, *c-Myc*, *Bcl2* and *MET*. Unexpectedly, none of the detected *miR-34c* targets were negatively regulated by E2F1 as *SCF* was (Figure 5B), indicating a possible specific effect of E2F1 on *miR-34c*-SCF axis in CRC cells.

**Figure 6:**
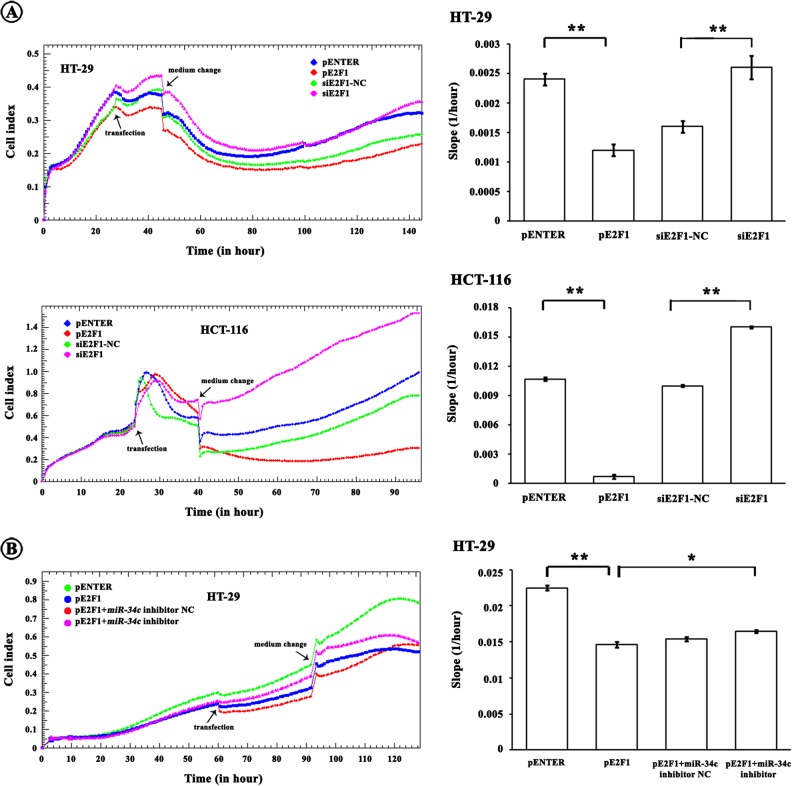
Involvement of *miR-34c* in E2F1-reduced cell proliferation (**A**) E2F1 suppressed CRC cell proliferation monitored by RTCA (*P<0.05, **P<0.01). (**B**) The E2F1-reduced proliferation was partially abrogated by an introduction of *miR-34c* inhibitor (*P<0.05, **P<0.01).

Corresponding to the SCF alterations, E2F1 over-expression caused proliferative suppression whereas hypo-expression of E2F1 speeded up proliferation (Figure 6A). To further investigate whether the increase in *miR-34c* was involved in the proliferative inhibition resulted from E2F1 over-expression, we performed a *miR-34c* loss-of-function experiment. The proliferative suppression by E2F1 was partially restored by an introduction of *miR-34c* inhibitor in HT-29 cells, suggesting that the E2F1-induced *miR-34c* contributed to the repression of cell proliferation (Figure 6B). We noted that the *miR-34c* inhibitor did not completely abolish the decreases of cell proliferation caused by E2F1, implying that *miR-34c* could not be the sole factor implicated in the E2F1-reduced proliferation and additional regulatory pathways must be involved.

### SCF/KIT signalling raises E2F1 expression

**Figure 7:**
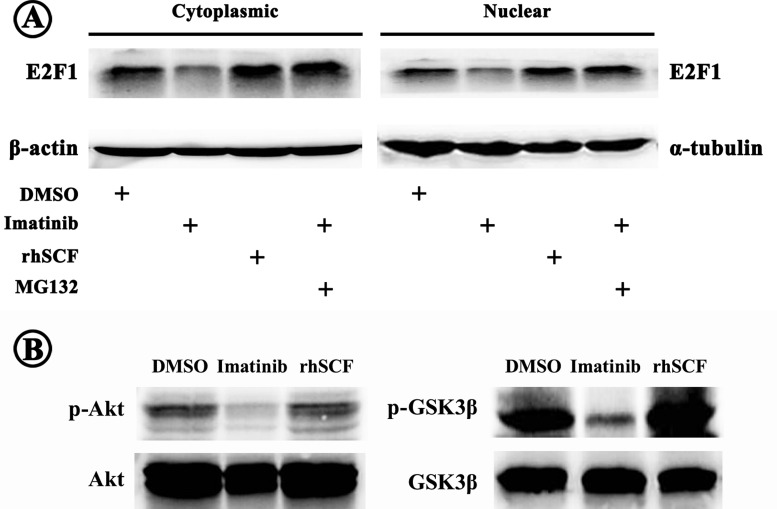
Regulation of SCF/KIT signalling on E2F1 expression (**A**) Activation or inactivation of SCF/KIT signalling resulted in increase or decrease in cytoplasmic and nuclear E2F1 expressions respectively. The decreased E2F1 was recovered by additional proteosomal inhibitor MG132. (**B**) SCF/KIT signalling activated its downstream Akt which further inactivated GSK3β through phosphorylation.

Aberrant SCF/KIT autocrine/paracrine stimulation loop plays a critical role in CRC development by activating various downstream effectors including PI3K/Akt, JAK/STAT, Ras/MAPK etc. which participate in multiple cellular functions. We proposed a theory that SCF/KIT may regulate E2F1 production and accordingly affect CRC cell bioactivities. Indeed, cytoplasmic and nuclear E2F1 levels were both raised in HT-29 cells over-expressing KIT in the presence of exogenous rhSCF (Figure 7A). On the contrary, E2F1 expression was attenuated when SCF/KIT signalling was blocked by Imatinib; and the reduced E2F1 was rescued by additional MG132, an inhibitor of proteosomal degradation (Figure 7A). We also observed decreased phosphorylation of Akt and spontaneous activation of GSK3β after exposure to Imatinib (Figure 7B). While, activating SCF/KIT signalling exerted opposite outcomes (Figure 7B). These results suggested the existence of E2F1-*miR-34c*-SCF negative feedback loop.

## DISCUSSION

Accumulating evidences have been suggesting a tumour suppressing role of *miR-34c* in CRC [4,6,12,14]. Majority of the previous documents focused on the targets of *miR-34c* in CRC. But little was known about the molecules that mediated *miR-34c* expression in CRC, except p53. However, it was inferred that p53 is not an exclusive regulator of *miR-34c*; and some other unknown factors especially those have ability to recruit to *miR-34c* promoter and promote its transcription are involved. In the present study, we demonstrated that, besides p53, E2F1 was a potent activator for *miR-34c* transcription in CRC cells. An E2F1-binding site (−897 to −889 bp region upstream of *pre-miR-34c*) was identified which was harboured within a hyper-methylated CpG island and hardly bound to E2F1, explaining the lower level of *miR-34c* in CRC tissues and cells. Over-expression of E2F1 induced *miR-34c* expression; on the contrary, knockdown of E2F1 exerted opposite effects. The rise of *miR-34c* by E2F1 was enhanced by an addition of DAC treatment, further verifying the E2F1-binding site in the *miR-34c* promoter and supporting the role of E2F1 in promoting *miR-34c* transcription.

**Figure 8:**
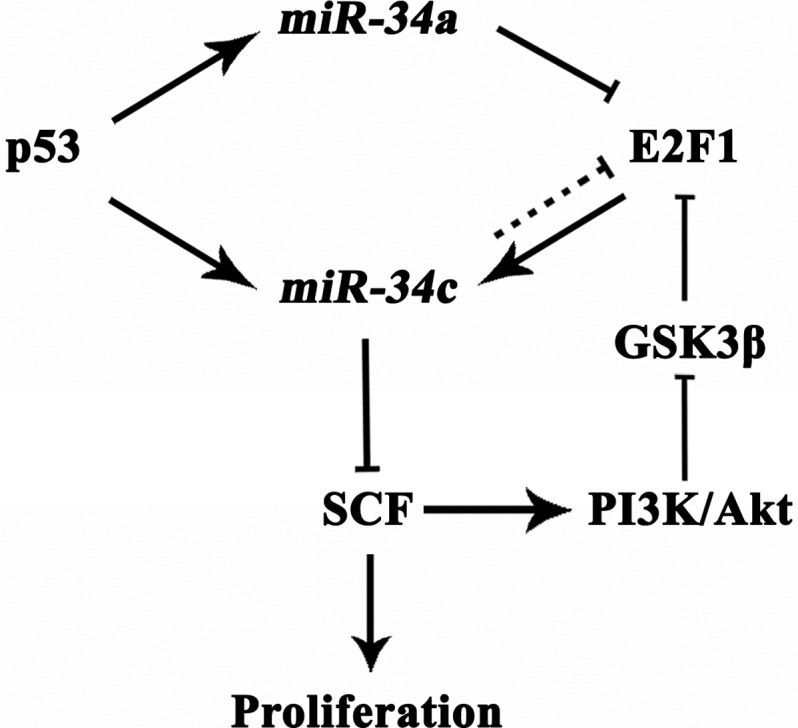
Model of regulatory loops among p53, *miR-34s*, E2F1 and SCF with a role in CRC proliferation.

E2F1 is the most deeply investigated member in the E2F family of transcription factors serving key roles in cell cycle, apoptosis, cell differentiation and stress responses. Although the functions of E2F1 in different cancers are in controversy, there is a consensus that E2F1 plays a tumour suppressing role in CRC [15–17]. For the past few decades, efforts have been paid on seeking for the targets of E2F1 that were implicated in carcinogenesis. One of the most intriguing outcomes was that through trans-activating ICAT, E2F1 could negatively regulate Wnt/β-catenin activity, whose aberrant activation contributes to cell growth, tissue invasion and metastasis of CRC [18,19]. Here, we addressed that the tumour suppressing *miR-34c* was probably another *bona fide* target of E2F1 and could be positively regulated by E2F1. The E2F1-increased *miR-34c* suppressed CRC cell proliferation, supporting a tumour suppressing role of E2F1 in CRC. Interestingly, *miR-34c* has been reported to silence some components of the canonical Wnt/β-catenin signalling cascades [20]. These literatures indicated a synergic effect of E2F1 and *miR-34c* in inhibiting tumour progression. However, Tazawa et al. [21] stated that *miR-34a* caused senescence-like growth arrest of CRC cells by silencing E2F1 and E2F3, leading to an accumulation of p53. This strongly argued that *miR-34s* strengthened the anti-proliferation activity of E2F1, but suggested a complicated mutual regulation between the E2F1 and *miR-34* family members. In agreement with Lize et al. [22] we presumed here that E2F1 was not only an important modulator of *miR-34c* expression, but also itself was regulated by *miR-34a/c* in an auto-regulatory feedback loop, which avoided an excessive E2F1-reduced proliferation (Figure 8).

With regard to the molecules involved in the E2F1-inhibited CRC cell proliferation through *miR-34c*, we examined some previously identified *miR-34c* targets. The results revealed that SCF, which triggers cell proliferation by stimulating its receptor KIT, was significantly down-regulated by the E2F1-induced *miR-34c*, indicating that knockdown of SCF was involved in the anti-proliferation by E2F1. Unexpectedly, except SCF, the rest *miR-34c* target genes (*Myb*, *c-Myc*, *Bcl2* and *MET*) did not show inverse correlation with *miR-34c* level. It seemed contradictory to previous studies; but by reviewing literatures, we learned of that E2F1 could directly and positively regulate *Bcl2*, *c-Myc* and *Myb* [23–25]. We proposed that the expressions of *Bcl2*, *c-Myc* and *Myb* were predominantly regulated by E2F1 through direct transcriptional regulation in CRC cells instead of indirect modulation through *miR-34c*. Our results provided a clue of a specific regulatory pathway of E2F1-*miR-34c*-SCF in CRC cells. Nevertheless, a recent publication demonstrated that E2F1 could recruit to *SCF* promoter and induce *SCF* expression in non-small cell lung cancer cells [26]. The possible explanations for the opposite results could be (1) The experiments by Perumal et al. [26] were conducted in lung cancer cells. It is well accepted that the contribution of E2F1 in carcinogenesis is tissue dependent. Though E2F1 played a tumour-suppressing role in CRC, its expression was usually increased in non-small cell lung cancer and associated with worse patient prognosis [27,28]. Therefore, it raised the possibility that the mediation of E2F1 in the SCF expression is also tissue specific. (2) As Perumal et al. [26] described, there were not maximal amounts of E2F1 associated with the SCF promoter in quiescent lung cancer cells until stimulated with nicotine, suggesting the binding of E2F1 to SCF promoter was in a context-dependent manner.

Furthermore, we showed that SCF/KIT signalling had a positive effect on E2F1 production. To investigate how SCF/KIT signalling mediated E2F1, we detected the downstream of SCF/KIT signalling, Akt and GSK3β, given that GSK3β could inhibit E2F1 by promoting its ubiquitination through physical interaction [29]. Consistently, our results showed that highly activated SCF/KIT signalling raised phosphorylated Akt which increased E2F1 by inactivating GSK3β. The reduced E2F1 by blocking SCF/KIT signalling was reversed by MG132, further validating that SCF/KIT signalling pathway regulated E2F1 expression via mediating its proteosomal degradation. Based on the results, we proposed a negative feedback loop consisting of E2F1, *miR-34c* and SCF, which could maintain intracellular homoeostasis under physiological state. Nevertheless, pathological hyper-methylation obstructed the recruitment of E2F1 to the *miR-34c* promoter leading to the interruption of the feedback loop and accordingly aberrant SCF/KIT signalling which promoted the development of CRC.

Notably, the E2F1-suppressed CRC cell proliferation was not exclusively through a *miR-34c*-dependent manner. There are likely other transcriptional targets of E2F1 involved. In this respect, *miR-449a/b* were identified as transcriptional targets of E2F1 [22]. *miR-449a/b* structurally resemble the *miR-34s* and display tumour-suppressing function by suppressing cell proliferation and promoting apoptosis of CRC cells [22]. Like *miR-34a/c*, *miR-449a/b* provided a negative feedback on E2F1 [22]. Furthermore, E2F1 trans-activated *miR-449a/b* which in turn activated the p53 pathway, thereby inducing *miR-34* expression [30], forming a net-work of p53, *miR-34s*, *miR-449s* and E2F1.

In the present study, we used *p53^+^* HT-29 cells and *p53^−^* HCT-116 cells; and both cells expressed increased *miR-34c* upon the over-expression of E2F1, suggesting that the effect of E2F1 on *miR-34c* transcription is p53 independent. Whereas, the transcription-promoting effect of E2F1 on *miR-34c* was pronouncedly augmented in *p53^+^* cells, indicating a potent synergic effect of p53 and E2F1 (Figure 8).

In conclusion, we demonstrated that E2F1 was a transcriptional activator for *miR-34c* which further knocked down its target SCF. The E2F1-induced *miR-34c* was enhanced in the presence of p53 and exposure to DAC. The inhibited SCF/KIT signalling activated the negative feedback loop through decreasing E2F1, which was benefit for the intracellular homoeostasis. Hyper-methylation of the *miR-34c* promoter interrupted the negative feedback loop which was probably responsible for the CRC cell proliferation. Indicative of previous findings and our present data, we suggested that p53, E2F1, *miR-34c* and SCF could make up a regulatory mesh-work allowing fine tuning of their responses (Figure 8) and these molecules could be promising therapeutic targets for CRC patients even those who have lost p53 function due to mutations.

## Abbreviations

ADR: adriamycin
CRC: colorectal cancer
DAC: 5-aza-2′-deoxycytidine
HRP: horseradish peroxidase
MSP: methylation-specific PCR
SCF: stem cell factor
TSS: transcriptional start site
